# Institutional inconsistencies and professionals’ hidden institutional work in Russian pandemic-affected healthcare: The material dimension

**DOI:** 10.1177/13634593241303620

**Published:** 2024-12-02

**Authors:** Ekaterina Borozdina, Anna Temkina

**Affiliations:** Tampere University, Finland; Ben-Gurion University of the Negev, Israel; Ruppin Academic Center, Israel

**Keywords:** ethnography, organization of health services, profession and professionalization

## Abstract

In recent years, medical sociology has produced a significant amount of publications about the effects of the COVID-19 pandemic on medical care provision and healthcare professionalism around the globe. This study builds on this line of research by looking at a rarely discussed case of pandemic management—the case of Russia’s centralized and state-dominated medical sector. In our analysis, we focus on the organizational level and the institutional work of front-line health professionals. Using a neoinstitutional theoretical lens, we show how, as a result of the conflict between professional and managerial logics, pockets of extreme institutional uncertainty emerged within Russian healthcare: “non-COVID” healthcare facilities and hospitals rapidly restructured for COVID-19 care. Qualitative interviews with healthcare professionals indicate that institutional misalignment inside these “gray zones” translated into the material dimension, significantly impeding the effectiveness of the pandemic response. While sociological literature frequently portrays Russian health professionals as fully subjected to administrative constraints and disempowered, our data allows us to trace their informal institutional work and agency during the health crisis. Through these materially mediated work, our informants attempted to deal with both the challenges of the pandemic and institutional contradictions of the Russian healthcare system. Professionals’ institutional work brought some improvements to Russia’s pandemic-affected clinical settings. However, being informal and purposefully hidden, it neither constituted a viable solution for medical organizations, nor contributed to the strengthening of professionals’ autonomy.

## Introduction

The COVID-19 pandemic presented a major challenge to healthcare systems around the globe. While the lack of protective equipment and insufficient medical knowledge about the new infection were immediately admitted as problems, recent studies increasingly draw attention to the broader issues of health policies and institutional arrangements in COVID-19 crisis management (e.g. Adams and Wannamaker, 2022; Shuster and Lubben, 2022). Contributing to this line of research, this article analyzes how institutional inconsistencies impeded the efficiency of the pandemic response in Russia’s highly centralized healthcare system. We use qualitative interview data to center on how those organizational challenges interacted with the materiality of clinical work and how front-line healthcare professionals met palpable institutional inconsistencies amidst the health crisis.

Russia experienced one of the most severe impacts of the pandemic among states with developed healthcare systems. By the end of 2021, the country had one of the world’s highest numbers of excess deaths due to COVID-19: 374.6 deaths per 100,000 of the population, and around 1.07 million deaths in total (Wang et al., 2022). Investigative journalists further argued that by May 2020, Russia had the highest COVID-19 mortality rate among healthcare practitioners worldwide (Litavrin et al., 2020). These facts contradict initial scholarly expectations, according to which Russia’s centralized health system—with universal access to medical services—should have been an advantage in any health emergency (Cook and Twigg, 2020).

Recent scholarly efforts to solve the puzzle of Russia’s inadequate pandemic response have concentrated on the macro level, exposing inconsistencies in Russian pandemic policies. Researchers describe these policies as a combination of technocracy, authoritarianism, and populist appeals (Shirikov et al., 2023). Specifically, they outline the contradictions between the compulsion-based state pandemic response that limited local initiative and sporadic attempts of the federal government to delegate pandemic management to regional authorities, who lacked resources to tackle the crisis (Åslund, 2020; Shirikov et al., 2023). By focusing on the organizational level and on health professionals daily routine, this study adds more nuance to our understanding of institutional inconsistencies that emerged in the country during the pandemic.

Conceptually, the article builds on neoinstitutional studies of healthcare. This approach explores how values, beliefs, and rule systems affect medicine as an organizational field (Scott et al., 2000). It also examines how medical workers navigate institutional arrangements, as well as the consequences of their agency for the quality of services and professional power (Currie and Spyridonidis, 2016). While neoinstitutional scholarship typically concentrates on ideational aspects of organizational life, recent publications suggest bringing to the fore the material dimension of institutions (Friedland and Arjaliès, 2021). This study answers this call by investigating the material dimension of both institutional logics and professionals’ institutional work in Russian pandemic-affected healthcare.

We consider Russian hospitals restructured for COVID-19 care and “non-COVID” medical facilities as a vivid example of the conflict between managerial and professional logics in the state-dominated health system in non-democratic regime. The study focuses on the tangible consequences of this misalignment for the work of organizations during the health crisis. While sociologists repeatedly portray Russian front-line healthcare professionals as subjugated to bureaucratic control and deprived of agency (Kuhlmann et al., 2019; Saks, 2015), we outline how institutional contradictions actually stimulated professionals’ institutional work. However, being materially mediated and intentionally hidden, this institutional work did not led to the increase of professionals’ authority and autonomy.

## Background

### An institutional logics approach in studies of healthcare professions and organizations

We employ the institutional logics approach to analyze the organizational implications of the COVID-19 pandemic, as well as the response of Russian health professionals to it. The studies of institutional logics constitute one of the main lines in current sociological research on healthcare. This concept is used to describe the sets of “material practices and symbolic constructions which constitute organizing principles and which are available for individuals and organizations to elaborate” (Friedland and Alford, 1991: 248). Logics bring order to organizational fields, including the medical field, by setting normative guidelines and frames of reference for institutions and their inhabitants (Thornton and Ocasio, 2008).

Regarding healthcare, scholars most commonly outline three ideal types of institutional logics: professional, managerial, and market logics. Professional logic prioritizes expert knowledge, the quality of medical services, and professional self-governance. Managerial logic foregrounds the value of equal access to services, administrative accountability, and managers’ control over work in a bureaucratic hierarchy. Market logic is linked to the principle of cost-efficiency, with market competition and customers’ preferences determining the design and offer of services (Scott et al., 2000: 166–235; Freidson, 2001). A plethora of studies have documented the structural transition of modern ’Western’ medicine from the dominance of professional logic to the era of managerial control and market mechanisms. This process coincided with subjugating professionals’ autonomy to governmental authorities, insurance companies, and healthcare customers (Scott et al., 2000: 21–22).

In subsequent research, this broad perspective on institutional changes has been supplemented by a deeper analysis of how individuals within organizations interpret and enact different logics (Currie and Spyridonidis, 2016; Hallett and Ventresca, 2006; Reay and Hinings, 2009). According to the inhabited institutionalism approach, the tensions between the logics inevitably stimulate the agency of ground-level actors, since the latter are compelled to address the misalignments in their daily routine (Seo and Creed, 2002). Thus, in addition to their job tasks, health professionals have to engage in another form of work—institutional work that is aimed at maintaining or transforming organizational arrangements (Suddaby and Viale, 2011). In doing such work, they negotiate, resist, or hybridize different logics (Correia and Denis, 2016; Currie et al., 2012; McCann et al., 2013; McGivern et al., 2015).

Notably, the literature on institutional logics and institutional work has predominantly centered on concepts, cognitive schemas, and sense-making. Writing about healthcare, authors typically consider how managerial and budgetary pressures challenge the medical profession’s dominant value of the quality of care, and how physicians engage in institutional work to maintain the primacy of this value (Berghout et al., 2018; Wright et al., 2017). Recent studies have increasingly questioned this approach for overlooking the tangible dimension of organizational life and have called for the more consistent incorporation of materiality into institutional research (De Vaujany et al., 2019; Jones et al., 2013). They draw attention to the fact that “an institutional logic is not a logical relation of propositions grounded in the presumption of representation; it is a productive logic of practice” (Friedland and Arjaliès, 2021: 46). Materiality contributes to the maintenance, change, and disruption of organizational arrangements. Thus, our understanding of how institutional logics pervade the fabric of organizations is insufficient unless we grasp how the logics are embedded in and enacted through physical objects and space (Lawrence and Dover, 2015; Svensson and Gluch, 2022).

This study expands upon this line of thought by outlining the tangible inconsistencies between professional and managerial logics in Russian healthcare during the COVID-19 pandemic. Our analysis focuses on health professionals’ perspective and on institutional work, which professionals were compelled to exercise in response to the institutional misalignments. Literature on institutional work, mostly related to ’Western’ societies, highlights how even on the ground level professionals’ efforts can result in the increase of professional status and autonomy (Currie et al., 2012; McGivern et al., 2015). Social studies of technologies also indicate that as health workers’ adapt new digital tools for organizations’ immediate tasks, they simultaneously widen their jurisdiction and increase their professional status (Håland, 2012; Kamp and Hansen, 2019). In this article, we combine the interest in materiality with attention to institutional context. We explore professionals’ materially mediated institutional work in a centralized healthcare system, as well as the consequences of this work for professionals’ autonomy and power.

### Professions and institutional logics in post-socialist healthcare

Historically, Russian medicine has not experienced an era of professional dominance. In the Soviet period, the state acted as the principal player in healthcare governance, intervening in such essential areas of professionalism as professional education, ethics, medical knowledge production, and the functioning of professional associations (Saks, 2015; Vasilyev et al., 2021). Since medical professionals lacked the political power necessary to argue their perspective, the health system mostly dealt with immediate tasks in a managerial way (Navarro, 1977: 113). After the dissolution of the Soviet Union, a series of reforms introduced the principles of neoliberal management into the Russian health system and stimulated its rapid marketization. However, with governmental bodies occupying the leading role in healthcare funding and regulation, centralized state control over the field has maintained (Cook, 2014).

While private medical services have developed in Russia, state healthcare organizations continue to employ the vast majority of physicians and nurses. Professionals’ work is regulated in a top-down manner through a chain of command, which includes the Russian Ministry of Health, its regional offices, and heads of facilities. Multiple state controlling bodies, for example, the Federal Mandatory Health Insurance Fund and the Investigative Committee (Public Prosecutor), monitor medical facilities’ adherence to a variety of rules and enforce professionals’ accountability. This marriage of statism and neoliberalism in health governance has further weakened the position of Russian medical workers. They find themselves at the bottom of the state bureaucratic structure, while facing managerial pressure to provide cost-effective services.

Moreover, in order to maintain universal access to healthcare, the Russian state tends to sacrifice the quality of medical services (Twigg, 2002). Studies have documented infrastructural breaks in state-funded healthcare: the limited availability of laboratory testing, broken medical equipment, geographically scattered clinical facilities, unrealistic work schedules for medical personnel, and poor coordination between different facilities (Kamenshchikova et al., 2021; Temkina et al., 2022). To navigate this volatile setting and ensure access to better treatment, Russian citizens often use paid solutions, which range from private health services to informal payments at state medical facilities. However, neoliberal reforms have been relatively successful in enhancing accountability in the country’s healthcare and promoting legal market options (Temkina and Rivkin-Fish, 2020).

Due to the minimal impact of Russian professional associations on health governance, professionals’ ability to improve the situation is mostly limited to tacit efforts of individual health workers or informal teams. Studies show that some doctors, nurses, and midwives are able to enhance their workplace autonomy as a result of this institutional work (Borozdina and Novkunskaya, 2022). Usually, these professionals introduce new ideas and approaches in their practice by relying on market logic, and offering new commercial services to wealthy clients.

In the following parts of the article, we focus on care provision during the COVID-19 health emergency. We explore how the contradictions between professional and managerial logics led to organizational uncertainty in Russian medical facilities, increasing the vulnerability of medical practitioners and reducing the quality of medical care provision. We draw attention to the materially mediated institutional work through which healthcare professionals tried to deal with these institutional inconsistencies.

## Methods

### Sampling

We used purposive maximum variation sampling, recruiting informants, who at the time of the COVID-19 pandemic worked in different types of Russian healthcare facilities and in different medical subfields. By doing so, we tried to gain insights into the diversity of the institutional consequences of and responses to the health crisis. Since access to the field was restricted not only by the emergency, but also by the governmental efforts to downplay the effects of the pandemic, we recruited our informants using a snowball method, finding the first informants among our social networks and later using contacts provided by them. To ensure a diverse range of professional experiences in our sample and to minimize bias, we began the formation of the sample with the contacts drawn from eight researchers. When recruiting responders, we utilized a maximum of three contacts suggested by the same person.

The final research sample comprises 51 interviews with 49 healthcare professionals (two informants were interviewed twice). It includes 26 doctors, 2 midwives, and 10 nurses who worked on the front-line, as well as 8 hospital administrators (physicians), and 3 chief nurses. The interviewees worked in such healthcare fields, as allergology, anesthesiology and resuscitation, cardiology, general practice, gerontology, gynecology and obstetrics, oncology, otolaryngology, clinical pathology, pediatrics, radiology, treatment of infectious diseases, and surgery. Their work experiences ranged from 1 to 36 years of work, with a median of 13.5 years of work. During the pandemic, 29 informants had experience of directly providing care for COVID-19 patients. The following types of state-funded medical facilities are presented in the sample: non-COVID hospitals (specialized hospitals, multispecialty hospitals, research hospitals); non-COVID outpatient clinics; infectious disease hospitals, dedicated COVID-19 hospitals, and hospitals repurposed for the admission of COVID-19 patients; ambulance services.

This sample composition imposes several limitations on the possibility of the generalization of the research results. First, our materials reflect only the experiences of Russian professionals who worked in state-funded medical facilities. Organizational arrangements in private clinics differed significantly from this picture, but they are not discussed in this article. Second, the majority of the research participants lived and worked in St. Petersburg (*N* = 40), six were from Moscow, and three were from smaller Russian cities. Thus, the study’s findings are mostly informative about the situation in Russia’s largest cities, where healthcare organizations usually have better funding, equipment, and access to educational opportunities. Finally, 40 out of 49 informants worked in hospitals, which means that this study does not provide much insight into pandemic-related challenges faced by professionals in outpatient clinics or other types of medical organizations.

### Research ethics

The project received ethical approval from the Ethical Committee of St. Petersburg Sociological Association. Before the interviews, participants were informed about the research goals and procedures, as well as about the ways in which the study material would be used. We obtained informants’ oral informed consent for participation and discussed with them their preferred method of interview recording. Four informants objected to voice recording, in which case, the interviewers created detailed text summaries of the talks. All research data was anonymized. In order to preserve the anonymity of the informants, in the text of the article they are identified by their clinical specialty and a randomly assigned numerical code. We conducted all the interviews prior to the regulatory changes that prohibited Russian health practitioners from spreading information about the pandemic. Therefore, the study did not expose the respondents to legal risks.

### Data collection

The interviews were collected in March–June, 2020. Due to epidemiological restrictions, all interviews were conducted remotely. The duration of the interviews ranged from 20 minutes to 2 hours, with the mean duration being 46 minutes. The interview guide covered a wide spectrum of themes, including pandemic-related regulatory changes and their implementation in medical facilities; development and dissemination of scientific knowledge about the coronavirus infection; professionals’ work experiences during the first wave of the pandemic and interaction with patients; professionals’ own health, emotions, and changes the pandemic brought to their private lives; additional pandemic-related challenges. Interviews were collected by the authors and a team of researchers under the authors’ supervision.

The interviews were conducted, transcribed, and analyzed in Russian. The authors, who are native Russian speakers, then translated selected fragments of the interviews to illustrate the arguments presented in the article.

### Data analysis

Thematic analysis was implemented to analyze the materials (Strauss and Corbin, 1998). First, the Atlas.ti software was used to code the full texts of interviews and interview summaries. During this phase, the first author created research memos to outline the particularities of each informant’s case, and preliminary analytic ideas. Second, the first author grouped the codes under five wider themes connecting these to the categories that guided the research design—regulatory changes, organization-level changes, medical knowledge about the new infection, health professionals’ experiences, and professionals’ strategies. At this stage, the differences between Russian medical facilities in terms of pandemic management stood out, in particular, the differences between specialized infectious disease hospitals, facilities that were rapidly converted into hospitals for COVID-19 patients, and those medical facilities that continued providing services to non-COVID patients during the pandemic. A second researcher reviewed the data and the coding. The analysis presented in this article reflects the intersection between this classification of medical facilities and themes that were discussed in the interviews.

## Organizational “gray zones” in Russian healthcare during the pandemic

### Political rationale behind Russia’s pandemic response

Policy researchers outline two issues that were characteristic to Russia’s pandemic response: (1) the prioritization of political rather than public health rationale; and (2) decentralization as a means of deflecting political responsibility (Åslund, 2020; Shirikov et al., 2023). During the pandemic’s first wave, President Putin focused on advancing constitutional reform that would extend the duration of his presidency. The authorities were hesitant to admit a health crisis existed since it interfered with the President’s plan for holding a nationwide referendum in support of the reform. In order to proceed with their political goals, the government tried to downplay the extent of the pandemic. They also intended to showcase their ability to successfully manage the emergency before the vote.

The country’s officials arguably manipulated COVID-19 mortality data (Karlinsky and Kobak, 2021) and prosecuted those who publicly criticized their actions. In April 2020, the article “Public dissemination of knowingly false information about circumstances that pose a threat to the life and safety of citizens,” was added to the Criminal Code. This law was meant to prevent critical discussion and the public voicing of pressing pandemic issues (Arkhipova and Peigin, 2021).

The federal authorities also tried to avoid political responsibility for pandemic-related measures (and blunders) by delegating a significant amount of decision-making to regional governments. This unexpected decentralization had an adverse effect on the pandemic response because regional authorities frequently lacked the necessary resources and relied on inaccurate data (Åslund, 2020).

As for healthcare professionals, during the pandemic, the state treated them with increased attention and remuneration. However, these were misbalanced in different parts of the health system and coupled with greater administrative surveillance. While medical workers welcomed the rise in financing, they criticized its uneven and unjust allocation among different regions and types of medical facilities. Sociologists also documented physicians’ discontent with the intensified intervention of state controlled bodies into hospital routine (Oslon et al., 2021). In October 2020, the Russian Ministry of Health prohibited the country’s health practitioners from spreading any information about the pandemic without the Ministry’s approval. Consequently, Russian health workers, who experienced excessive workload and burnout due to policy inefficiency (Rozhdestvenskiy et al., 2022), had few options to influence decision-making or raise their concerns publicly.

### Organizational “gray zones” in pandemic-affected healthcare

As the shock of COVID-19 reverberated throughout the Russian health system, different parts of this organizational field experienced the pandemic quite differently. One might anticipate that medical facilities whose primary mission was the treatment of infectious diseases—the infectious disease hospitals—would have suffered the most from the pandemic. However, our informants refuted this view. They identified two types of Russian healthcare facilities that faced the most challenges throughout the health crisis: (1) facilities that continued the provision of regular medical help to non-COVID patients, and (2) facilities that were urgently restructured into COVID-19 hospitals after the pandemic strike.

While infectious disease hospitals served as a platform for the government to highlight its achievements in combating the coronavirus, non-COVID healthcare facilities were unsuitable for attaining this political objective. Consequently, facilities of this type fell out of both the authorities’ focus and the allocation of budgetary resources. On an administrative level, they were labeled as “clean” of coronavirus infection. This categorization severely limited these organizations’ ability to cope with the pandemic challenge in terms of epidemiological restrictions, infrastructural and material adjustments, and personnel schedule restructuring.

Medical facilities that were haphazardly converted into COVID-19 hospitals encountered their own set of challenges, primarily related to the urgency of the transformation. These medical centers had to completely reorganize their work routines, change the workspace and work schedules, and acquire new protocols for dealing with contagious patients. New material and spatial arrangements had to be created at once. The informants, who found themselves in the midst of these changes, complained about the low quality of transformation that could be accomplished in such a short time. For instance, informants reported that medical facilities started to admit patients with COVID-19 before sanitary checkpoints were fully prepared and able to work in full capacity. Because of this, ambulance cars had to wait in hours-long queues when delivering patients to hospitals.

At first glance, hospitals that were rapidly converted for COVID-19 treatment and “clean” medical facilities seem to be two distinct cases. However, they had a common problem: a severe discrepancy between the administrative categories they were placed in, which implied specific treatment requirements, and the actual medical care that health workers were able to deliver in given circumstances. The interviewees expressed awareness of the dangers that this situation posed for the patients.

Doctors in these [“clean”] hospitals are suffering from the coronavirus infection in the same way [as in the dedicated COVID-19 hospitals]. For older people, a trip to such a hospital, in principle, may end up being a one-way trip. (anesthesiologist-resuscitator_33)

After our informants, we describe both of these cases as organizational “gray zones” characterized by major contradictions between professional and managerial logics within the centralized and state-dominated healthcare system. In what follows, we elaborate on how this inconsistency of logics manifested on the material level of hospital routine and trace professionals’ attempts to mend the situation.

### Inconsistency between professional and managerial logics: The material dimension

Interview data allows us to distinguish three ways in which the decoupling of administrative classifications and professional logics were tangibly manifested in identified pockets of organizational uncertainty: (1) insufficient testing for COVID-19 in “clean” medical facilities; (2) the lack of personal protective equipment (PPE) in “clean” medical facilities; and (3) inadequate zoning of hospitals that were rapidly repurposed for the COVID-19 treatment. Below, we describe each case in more detail.

### Insufficient testing of medical practitioners for COVID-19

According to the informants, one of the major problems they faced during the pandemic was the insufficient screening of medical workers for coronavirus. This problem stemmed from a general shortage of test kits at that time. However, regulatory factors made the issue worse at “non-COVID” facilities. The rigid managerial classification resulted in the denial of the virus’s presence in such facilities. Healthcare professionals who worked there indicated that their administrations did not want to check the personnel for infection since the test results might jeopardize the official image of the “non-COVID” facility.

The hospital is “clean,” well, relatively “clean” - no one takes PCR tests here, so no one detects the coronavirus. Just like that. (anesthesiologist-resuscitator_17)

At the beginning of the pandemic, the Russian system of COVID-19 testing was monopolized and tightly controlled by the Federal Service for Consumer Rights Protection (the Rospotrebnadzor). This excessive centralization of screening created bottlenecks, leading to limited access to testing and delays for healthcare facilities across the country in receiving test results. The Rospotrebnadzor enforced a managerial vision of the pandemic that differed from the actual situation on the ground. Perceiving some medical facilities as “clean” from the coronavirus, Rospotrebnadzor drastically reduced the amount of COVID-19 test kits distributed to them. In the centralized Russian health system, professionals were unable to question higher-level directives, including the incorrect labeling of their workplaces as not exposed to the infection. Hospital administration also refrained from challenging the authorities.

The Rospotrebnadzor is in charge of this whole topic [of COVID-19]. This is a separate, completely bureaucratic organization, and all these tests are controlled by them (. . .) According to our medical officer, they [Rospotrebnadzor] gave us, like, ten tests for the whole hospital. And we have roughly one thousand two hundred employees. Of course, they [the hospital administration] could, I don’t know, purchase [more test kits], but this would be a very bold move, not typical for our administration. They are subjugated to the Rospotrebnadzor. (cardiologist_16)

Our informants pointed out that the Rospotrebnadzor’s perspective on the pandemic contradicted not only professionals’ actual experience but also scientific evidence. Doctors highlighted that the logic of Russian administrative bodies aligned neither with hospitals’ pragmatic needs nor with the very core of professionalism—the medical knowledge.

Despite what was taught [at medical universities]. . . Global [medical] practice says one thing, but nevertheless, Rospotrebnadzor makes its own decisions and imposes its own diagnoses. (infectious disease doctor_22)

### The lack of personal protective equipment within medical facilities

PPE was another crucial object during the first wave of the pandemic. In the Russian case, shortage of PPE was dramatically exacerbated by the strict distinction between COVID and non-COVID facilities. When administrative bodies labeled a medical facility as “clean,” they obtained a legal excuse not to deal with the shortage of PPE there. The quote below illustrates how employees in a non-COVID hospital, while daily encountering possibly infected patients, did not have the necessary means to protect themselves against contamination.

At the department, we don’t have the necessary PPE. This is all motivated by the fact that. . . It is legally justified. It’s legal since we are “clean” [non-COVID hospital], so we don’t need it. We [according to the regulations] don’t need this PPE. (cardiologist_19)

Some informants stressed that the issue was not just the shortage of PPE, but also that the distribution of limited resources was sometimes irrational and did not correspond to their job duties. For instance, employees of non-COVID hospitals received full-body protective suits, which were vitally needed in infectious disease wards. For general practitioners or cardiologists, disposable masks and gloves would be more important, but they did not receive them.

Notably, administrators at the organizational level recognized the significance of staff protection against the virus, but resorted to managerial ways of dealing with the problem. They required front-line professionals to wear PPE while on the hospital’s premises and to sign corresponding written consent. Administrators inspected workplaces on a regular basis to ensure personnel followed those rules. However, because the real reason for inadequate PPE supply had not been addressed, all of these solutions were merely superficial. They only exacerbated the problem: under administrative pressure, healthcare professionals had to reuse disposable PPE, further endangering their own and patients’ safety.

A: We must, naturally, be in [personal] protective equipment. We are very harshly criticized if we are not. There are a lot of [administrative] inspections quite often (. . .) We must wear special protective screens, [we must wear] such robes on a robe.

Q: Are you provided with them?

A: Well, that’s another question. Personally, I give [protective] masks to doctors and nurses in the morning—one for the day. These are ordinary masks, which you are supposed to use for 2 hours (. . .) Some people wear them for 3 days. (oncology nurse_1)

### Inadequate “decorative” zoning of hospitals

During the pandemic, medical facilities were required to establish sanitary checkpoints and separate clean (“green”) areas from contaminated (“red”) areas, implementing increased safety protocols in the latter. However, informants reported that similar to other problems, zoning was conducted superficially. It was meant to appease potential administrative inspections rather than improve the safety of medical personnel and patients. In some medical facilities, effective zoning was even technically unattainable due to the construction characteristics of the building. A doctor, whose workplace was located in a 19th-century mansion, described the challenges that front-line professionals faced when the city authorities unilaterally decided to convert their hospital for COVID-19 treatment.

An infectious diseases hospital implies zoning of premises at a minimum. If your premises are not initially ready for this, you need to do a lot of work figuring out how to zone the room. There should be clean areas, dirty areas, and so on. This did not happen at [the name of the facility]. This didn’t happen not because everyone is scum and doesn’t want to do it, but because it is virtually impossible. (anesthesiologist-resuscitator_6)

In other healthcare facilities, the administration, not having the time and resources to do a thorough job of zoning, only pretended to divide hospital space into “green” and “red” zones. The refurbishment usually took place weeks after patients with coronavirus arrived at a supposedly “clean” hospital, and they were already housed in wards among non-infected patients without any protection. One of our informants called such zoning “a decoration” since it did not serve as an actual safety measure. She highlighted that this “decoration” even increased the risk of spreading COVID-19 in the facility, for example, by causing the overcrowding of personnel in locker rooms.

Everything has already been dirty [infected with coronavirus]. And then they [the hospital administration] (. . .) built a sanitary checkpoint and said that what was dirty before is now clean [of coronavirus]. Well, ok. . . The sanitary checkpoint itself is just a decoration. It was our locker room. It has been divided into two parts (. . .) They have installed [there] two shower cabins that leak and don’t have screens. And they have choked up the shower cabin that we actually used, which was working. This space is so cramped that you can hardly enter. I used to take a shower at work, but I just can’t do it now. (nurse receptionist_31)

The informants coined the “gray zone” metaphor to describe this discrepancy between the declared zoning of a hospital and the actual permeability of the boundaries to the virus. They utilized this metaphor to characterize hospitals’ spaces, the state of which was not clear in terms of contamination. “Gray zone” also alluded to the enormous organizational uncertainty during the pandemic and, as we elaborate later, personnel’s informal institutional work in response to inconsistent managerial regulations and their material consequences.

I work in a hospital, which. . . It was decided to make it a non-COVID [hospital]—a hospital in which there is no COVID. Well, of course it is here (. . .) Here we have no red zone, nor yellow, nor green, but everything is just a gray [zone]. (cardiologist_16)

## Professionals’ materially mediated institutional work in mitigating organizational inconsistencies

We argue that Russian healthcare practitioners had to perform informal institutional work coping with a conflict between managerial logic and their professional requirements to uphold the treatment quality. Due to their disempowerment within the centralized healthcare structure, professionals lacked formal opportunities to address those contradictions. Although the topic of inconsistencies was frequently brought up in the interviews, in only one case did medical staff effectively advocate a professional perspective. That was the case of a hospital in St. Petersburg that converted to treating COVID-19 patients despite a serious lack of PPE, oxygen, and medications. While the hospital administration ignored staff objections to the transformation, front-line employees organized collectively, appealing to the city governor, and launching a public discussion in local media.

Most health professionals, however, dealt with organizational materially embedded uncertainty not through explicit collective action, but through tacit ground-level efforts. We consider these professionals’ efforts as a form of institutional work since they helped Russian medical facilities cope with regulatory inconsistencies and delivering of services during the pandemic. We identify two interrelated components of such work: (1) adjusting for material shortages by making, mending, or purchasing necessary “objects” (PPE) and (2) by developing alternative protocols and informally zoning hospital space.

When our informants’ addressed the acute lack of PPE in potentially contaminated medical facilities they bought these essentials at their own expense or with the help of sponsors. Usually, they did this secretly, trying to reduce the risks of legal prosecution for revealing the presence of the virus in “clean” hospitals and demands for additional protection. For instance, one interviewee (midwife_8) recalled crowd-funding among acquaintances for PPE and then delivering the purchases to her workplace by personal car during the night to avoid being noticed. Other interviewees talked about stitching self-made protective masks from medical gauze or using snorkeling masks and painter’s overalls. The quote below offers the typical story of an informant from a supposedly “clean” facility who responded to the organizational ambiguity by creating her own PPE.

Neither I, nor anyone knew that we would begin to work with coronavirus [patients]. It was the end of March, and I just decided for myself that I don’t know what kind of patient will come to me. Since it is everywhere, this coronavirus, I decided to protect myself. That is, I bought respirators, my husband bought [for me] this painter overall. (radiologist_49)

Another form of materially enacted institutional work was linked to re-labeling the organizational “gray zones” as not “clean” and developing alternative protocols of operating inside these spaces. Our interviewees neither relied on official managerial classifications nor attempted to challenge them formally. Instead, professionals informally categorized patients and hospital areas based on their likelihood of being infected with COVID-19. They created alternative working procedures to mitigate the pandemic’s hazards in those areas. The quote below indicates how in one of the “clean” facilities, the intensive care department devised its “protocols” for dealing with potentially infected patients. The “protocols” were materially enacted through professionals’ institutional work. The entire department space was designated as high-risk, and doctors switched to using PPE when dealing with all patients. Notably, these changes were introduced rather tacitly: the interviewees did not discuss or coordinate their efforts even with the colleagues from the neighboring subdivisions.

We had established a system of infectious diseases approach to everyone, we considered absolutely all the patients who came to us for whatever reason as COVID+ (. . .) The surgeons among themselves also were dividing them [the patients] contingently. Since I didn’t understand their system, we simply considered everyone as COVID+ and treated them as if they were COVID+. That means appropriate protection, appropriate disinfection, and appropriate approach. There was just no treatment, since these patients. . . Once again, we did not [officially] treat [patients with] COVID (anesthesiologist-resuscitator_6)

In sum, during the pandemic Russian front-line health professionals were somewhat compelled to engage in materially mediated institutional work. However, their ability to mitigate institutional inconsistencies through these informal initiatives was limited. In the highly centralized and bureacratized system, doctors’ and nurses’ dispersed actions served as emergency measures rather than a deliberate attempt to influence institutional arrangements. Being consciously hidden, professionals’ institutional work led neither to sustainable improvement of care provision nor to an increase in professionals’ autonomy and power. On an organizational level, a mixture of formal regulations and alternative “protocols” frequently added to institutional ambiguity, resulting in inconsistencies between hospital departments as well as care discontinuity.

## Discussion and conclusion

A number of studies have already used qualitative methodology to describe how medical workers in different countries, including former Soviet republics, perceived and addressed the COVID-19 challenge (e.g. Foster et al., 2024; Rao et al., 2021). Our study contributes to this literature, particularly to the strand that focuses on institutional factors—pre-existing health systems’ weaknesses and inconsistent regulations,—which conditioned the flaws in pandemic response (Buchberger et al., 2024). Relying on interviews with Russian healthcare professionals, we outline (1) institutional discrepancies in the country’s healthcare during the COVID-19 crisis and (2) informal institutional work, which doctors and nurses performed in order to mitigate these discrepancies.

At the center of this study are two types of Russian state-funded healthcare facilities: non-COVID medical hospitals and hospitals repurposed for COVID-19 treatment. During the health crisis, these facilities experienced extreme organizational uncertainty because their new status was assigned administratively with little regard for the situation on the ground or medical science assumptions. This reflected a mismatch between managerial and professional logics in Russian healthcare, a mismatch that translated into the material dimension, hindering effective care provision and raising the risk of infection for both health workers and patients. Our interviewees lacked proper virus protection, were unsure of their or their patients’ infection status, and complained about inadequate zoning of hospital space. They coined the ’gray zone’ metaphor to describe the lack of certainty regarding what and who is contaminated with the coronovirus. This metaphor also revealed professionals’ confusion about which logic to follow: an administratively imposed managerial logic that did not correspond to their immediate workplace experience or a professional logic that prioritized clinical issues but was somewhat ignored by the governing bodies.

Russian healthcare professionals, disempowered at the structural level (Saks, 2015), rarely had institutional opportunities to challenge the regulatory bodies or voice their concerns publicly. In dealing with the pandemic, the authorities generally disregarded and silenced professionals’ opinions, prioritizing political goals and sweeping many problems in medical care delivery under the rug (Karlinsky and Kobak, 2021; Shirikov et al., 2023). Our informants, who found themselves in an organizational double bind—silenced, facing significant health risks, and not being able to perform professional duties as they saw fit—tried to informally mitigate institutional inconsistencies through ground-level efforts. The interviewees did not identify these activities as an intentional attempt to change institutional arrangements. However, we consider them an example of institutional work, which helped Russian health organizations in dealing with both the pandemic and regulatory challenges.

These findings add to the discussion on the grassroots agency in state-dominated health system under non-democratic political regimes, of which Russian case is an example (Temkina et al., 2022; Kamenshchikova et al., 2021; Nikulkin and Zvonareva, 2024). While sociological studies tend to portray Russian front-line doctors and nurses as deprived of initiative and fully subordinated to administrative control (Kuhlmann et al., 2019; Saks, 2015), we indicate a more complex dynamic. Since, during the pandemic, managerial logic starkly contradicted professional logic, creating risks for both medical workers and their patients, our interviewees were compelled to engage in institutional work. However, rather than challenging the established organizational order, this work aimed to subtly tinker with it. Front-line professionals usually refrained from openly criticizing the authorities due to the fear of legal prosecution. Consequently, their institutional efforts took an informal, hidden, and materially mediated form.

The analysis of this hidden institutional work contributes to the debate about the material dimension of institutions, which is gaining momentum in neoinstitutional research (De Vaujany et al., 2019; Friedland and Arjaliès, 2021). Studies conducted in the “Western” healthcare systems highlight that professionals can improve their status via institutional work, including the work, which is linked to the materiality of organizational life. For instance, when nurses routinely adjust hospital digital infrastructures to the reality of clinical practice, they gain more power and autonomy within medical organizations (Håland, 2012; Kamp and Hansen, 2019). Our research results show that the translation of professionals’ institutional work into a strengthening of their position is context-dependent rather than universal. Particularly so when we consider non-articulated and materially mediated institutional work, which can easily go unnoticed.

In the Russian state-controlled and bureaucratized healthcare, professionals’ informal institutional work, served as a coping or even survival strategy. It neither brought changes on the level of rules and regulations, nor contributed to the empowerment of the medical workers. The predominantly material form of this work allowed doctors and nurses to purposefully hide it from the controlling bodies, thus avoiding potential punishment. While this institutional work helped professionals in dealing with immediate difficulties, it neither provided a comprehensive solution to institutional inconsistencies nor led to sustainable improvements in medical care.
